# Optimizing therapy for del(17p) multiple myeloma

**DOI:** 10.18632/oncotarget.22987

**Published:** 2017-12-06

**Authors:** Hervé Avet-Loiseau, Paul G. Richardson, Alessandra di Bacco

**Affiliations:** University Cancer Center of Toulouse Institut National de la Santé, Toulouse, France

**Keywords:** multiple myeloma, optimal therapy, del(17p), high-risk cytogenetics

We read with interest the publication by Liu et al. reporting the results from a meta-analysis of 13 prospective studies evaluating the efficacy of proteasome inhibitor/immunomodulatory drug-based treatments for newly diagnosed (ND) and relapsed/refractory (RR) multiple myeloma (MM) patients with chromosome 17p deletion [del(17p)] [1]. In patients with RRMM, progression-free survival (PFS) was evaluated in seven trials including 1,419 patients, of which 393 had del(17p). The authors state that the PFS of del(17p) RRMM patients treated with single-agent dexamethasone, lenalidomide+dexamethasone (Rd) or bortezomib+Rd, carfilzomib+Rd, ixazomib+Rd (IRd), elotuzumab+Rd (ERd), or pomalidomide+dexamethasone, was: 1.1, 2–14.9, 24.5, 15.7, 21.2, and 4.6–7.3 months, respectively [1]. Based on these results, the authors suggest that combined treatment with carfilzomib or elotuzumab and Rd, or pomalidomide with low-dose dexamethasone, improves the outcomes of RRMM patients with del(17p) [1].

The meta-analysis of Liu et al. included efficacy data from the phase 3 TOURMALINE-MM1 study, which demonstrated a 35% improvement in PFS with IRd, compared with placebo-Rd, in 722 RRMM patients [2]. We would like to point out that in a pre-specified analysis of PFS in 69 RRMM patients with del(17p), median PFS was 21.4 months with IRd (compared with 9.7 months with placebo-Rd) [3], not 15.7 months, as reported by Liu et al. [1]. In TOURMALINE-MM1, all cytogenetic analyses were performed at a central laboratory, hence a cut-off value of 5% was appropriately used for defining the presence of the del(17p) genetic abnormality, based on the false-positive rate (technical cut-off) of the fluorescence in situ hybridization (FISH) probes used [3]. Subsequent post-hoc analyses demonstrated that the PFS benefit with IRd vs placebo-Rd was consistent when using different cut-off values for defining the fraction of cells with del(17p); the median PFS in the IRd (vs placebo-Rd) group was 21.4 months (vs 9.7 and 6.7 months) with a cut-off of 5% and 20%, respectively, and 15.7 months (vs 5.1 months) with a cut-off of 60% [3].

At present, there is no consensus on the appropriate FISH-positivity cut-off value for defining the presence of del(17p) and the International Myeloma Working Group recommends that no specific global cut-off should be applied [4, 5]. Currently, it is not known what minimum percentage of del(17p)-positive cells is associated with a poor prognosis, or whether this percentage varies depending on the treatment regimen/disease stage [5]. Most recent studies in MM patients with high-risk cytogenetic abnormalities have used a range of different cut-off values, from a single cell [6] to a threshold of ≥60% of cells [7], and it is therefore challenging to draw a link between the size of the pool of del(17p)-positive cells and its impact on clinical outcomes. Therefore, in our opinion, the meta-analysis from Liu et al. should report that the IRd PFS of del(17p) RRMM patients is 21.4 months, based on the protocol-specified cut-off value of 5% for del(17p) FISH-positivity, and not 15.7 months (based on a 60% cut-off). Furthermore, Liu et al. did not apply the same threshold when reporting outcomes for other clinical trials, such as the Lonial et al. 2016 study of ERd, which used a cut-off as low as a single positive cell [1, 6].

While we appreciate the authors' efforts in conducting the reported meta-analysis [1], we would like to highlight a major limitation of this study. Cross-trial comparisons have intrinsic limitations: the trials included in the meta-analysis differ in many ways, including, but not limited to, the number of patients, inclusion/exclusion criteria, treatment duration, and frequency of assessment. Furthermore, while the authors applied the 60% cut-off value to identify patients with del(17p), this definition has not been applied equally to all treatment regimens analyzed in the study. As such, no significant conclusions can be made on the relative impact of the different treatment combinations on the outcomes of RRMM patients with del(17p).

In summary, we would like to suggest to Liu and colleagues that, in light of our considerations, they re-evaluate the PFS results reported in their meta-analysis; in our opinion, the IRd PFS in del(17p) RRMM patients from the TOURMALINE-MM1 study should be corrected to 21.4 months.
